# Are inner nuclear layer ischemic lesions hidden indicators of retinal vein occlusion risk? A case-control study

**DOI:** 10.1186/s40942-023-00479-4

**Published:** 2023-07-05

**Authors:** Ramin Nourinia, Seyed Mohammadjavad Mashhadi, Seyed-Hossein Abtahi, Hosein Nouri

**Affiliations:** 1 Ophthalmic Research Center, Research Institute for Ophthalmology and Vision Science, Shahid Beheshti University of Medical Sciences, Tehran, Iran; 2 Labbafinejad Medical Center, Shahid Beheshti University of Medical Sciences, Tehran, Iran; 3 School of Medicine, Isfahan University of Medical Sciences, Isfahan, Iran

**Keywords:** Central retinal vein occlusion, Branch retinal vein occlusion, Optical coherence tomography, Paracentral acute middle maculopathy, Retinal ischemia

## Abstract

**Purpose:**

To investigate the association between retinal vein occlusion (RVO) and evidence of previous, unnoticed inner nuclear layer (INL) infarction in the fellow eye.

**Methods:**

This prospective case-control study compared the prevalence of INL lesions in the fellow eye of consecutive people with hypertension (PwHTN) with unilateral RVO versus a randomly chosen eye of an age-matched control group of PwHTN without RVO. En face slabs above the outer plexiform layer (OPL) were generated from 6 × 6 fovea-centered optical coherence tomography scans. Cross-sectional scans and en face slabs were surveyed for evidence of active/resolved ischemic INL lesions.

**Results:**

69 PwHTN were included and assigned to two groups, i.e., the RVO group (n = 37; 22 BRVO and 15 CRVO) and the control group (n = 32). There was no inter-group difference regarding age, gender distribution, and background diseases. Resolved INL lesions were more frequent in the RVO group (n = 26) than in the control group (n = 4) (70.3% vs. 12.5%, p < 0.001). BRVO and CRVO cases had similar prevalence of INL lesions in their fellow eyes. Unlike diabetes, ischemic heart disease, and gender, INL lesions were associated with RVO (in the fellow eye) with an odds ratio of 15.7 (95%CI: 4.17–76.73, p < 0.001).

**Conclusion:**

We identified a substantially higher prevalence of INL lesions in PwHTN with RVO compared to PwHTN without RVO. The atrophic appearance of lesions suggests they may serve as early markers of increased RVO risk in individuals with systemic or cardiovascular predisposing factors.

## Introduction

Retinal vein occlusion (RVO) is a common vascular cause of visual impairment or loss. The two major types are central retinal vein occlusion (CRVO) and branch retinal vein occlusion (BRVO) [1]. Precipitating factors may be ocular, such as glaucoma, or systemic, including aging, hypertension (HTN), stroke, ischemic heart disease (IHD), dyslipidemia, diabetes mellitus (DM), and smoking [1]. People with acute RVO may be asymptomatic or experiencing a spectrum of visual symptoms. Fluorescein angiography and optical coherence tomography (OCT) are often employed for diagnosis confirmation, risk stratification for neovascular complications, and monitoring of macular edema [2]. OCT may also reveal inner nuclear layer (INL) ischemic findings, including acute macular neuroretinopathy (AMN) and paracentral acute middle maculopathy (PAMM) [2].

PAMM is characterized by hyperreflective band-like lesions, indicating INL infarction [3]. Variations in severity, progression, and duration of retinal hypoperfusion give rise to a complex spectrum called retinal ischemic cascade [3, 4]. With enough time elapsed, unnoticed PAMM lesions become atrophic and leave foci of INL thinning, called “resolved” PAMM, which may be an early marker of a higher cardiovascular risk profile [5] – thus, the suggested term “*forme fruste*” retinal ischemic cascade [3].

Screening the retina for INL ischemic lesions in people with cardiovascular risk factors can help identify those at higher risk of retinal occlusive events and vision loss. This study explored and compared the prevalence of INL lesions in: (i) the contralateral eye of patients with HTN and acute RVO and (ii) people with HTN (PwHTN) with no history of retinal vascular pathologies.

## Methods

This prospective observational case-control study was carried out at Labbafinejad Medical Center, Tehran, Iran, from August 2021 to December 2022. The study protocol was approved by the Ethics Committee of Shahid Beheshti University of Medical Sciences (Tehran, Iran) (code: IR.SBMU.MSP.REC.1402.082).

Consecutive patients who visited our center with acute unilateral BRVO or CRVO who had underlying systemic HTN were included (the RVO group), in addition to an age-matched control group of individuals with HTN and no history of retinal vascular occlusive events (the control group). The ratio of controls to cases was 1:1. The study subjects were the contralateral eye of people with RVO and one randomly chosen eye of each person from the control group. We compared the prevalence of INL lesions – i.e., acute or resolved PAMM and AMN – between the two groups. We obtained self-reported history regarding HTN and confirmed it by reviewing the patients’ medical records or medications.

We excluded individuals younger than 40, monocular patients, and those with a history of hyperviscosity or any other disease involving the posterior segment, including diabetic retinopathy, glaucoma, and retinal artery occlusion. In addition, patients with a history of vitreoretinal surgeries, complicated cataract surgery, or penetrating globe injuries were excluded. So were eyes with opaque media, interfering with OCT acquisition and interpretation.

We documented participants’ demographic information, self-reported medical history, and RVO type. All included eyes underwent comprehensive ophthalmic examinations, including visual acuity (VA) assessment (via Snellen chart), anterior segment evaluation (on slit lamp examination), tonometry (Goldmann tonometer), detailed fundus examination (using a non-contact 78 D lens), and Spectral Domain OCT imaging (Spectralis SD-OCT, Heidelberg Engineering, Heidelberg, Germany) with a 6 × 6 mm macular cube scanning protocol. En face slabs at the level of INL were generated from 6 × 6 mm fovea-centered 3D scans of each included eye. Slabs were constructed by the built-in algorithm of the device, using two segmentation borders, namely the inner border of the outer plexiform layer and 50 microns above. Slabs were then evaluated by an experienced retina specialist (R.N) for hyper- and hyporeflective areas, corresponding respectively to regions of present ischemia (acute PAMM or AMN) and INL thinning (resolved PAMM). In cases of significant artifactual input, poor scan quality (signal strength < 6), or segmentation failure, OCT images were excluded.

Descriptive summary of numerical (mean and SD, or median and range) and categorical (frequency and percentage) variables were generated and used to compare (I) RVO cases vs. controls and (II) eyes with vs. without INL lesions, by Welch’s two-sample t-test (or Mann-Whitney U test) and χ^2^ test. We used Fisher’s exact test to evaluate the association between RVO and relevant variables. Normality of distribution of numerical variables was assessed by Shapiro-Wilk test – age was the only variable with normal distribution. Statistical data analysis was performed using R statistical software version 4.1.2 (R Foundation for Statistical Computing, Vienna, Austria) and the following open source packages: gtsummary [6] and ggplot2.

## Results

**Fig. 1:**
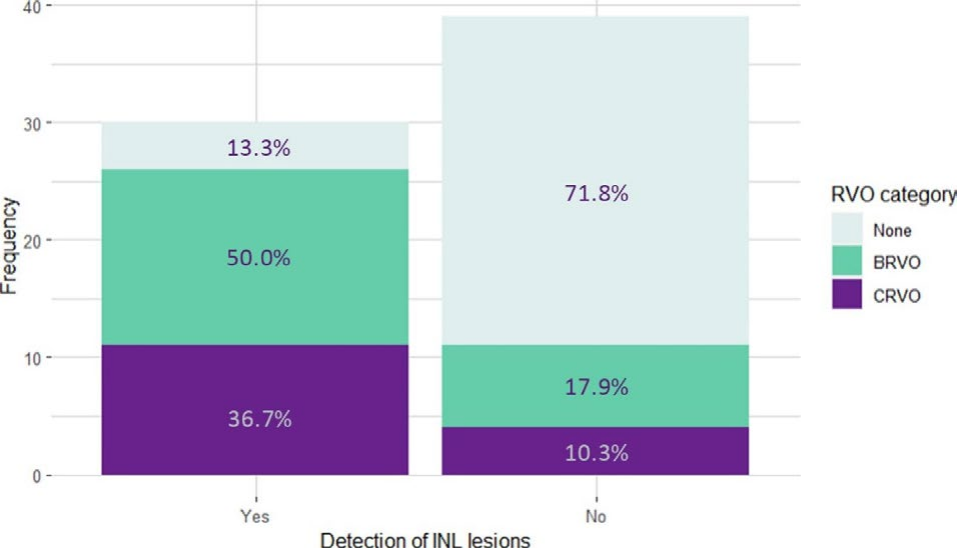
Bar plots illustrating stacked frequencies of retinal vein occlusion occurrence/type among individuals with and without evidence of previous inner nuclear layer lesions on optical coherence tomography

A total of 69 PwHTN were included. The RVO group consisted of fellow eyes from 37 RVO cases (22 BRVO and 15 CRVO), and the control group included 32 randomly chosen eyes from 32 control subjects. Table 1 presents the age and gender distribution in each group, their relevant medical history, visual acuity (logMAR), and the presence of INL lesions in their retinas (Table 1). The two groups were not significantly different regarding their mean age, gender distribution, and background diseases. INL lesions were more frequently detected in the RVO group (n = 26; resolved PAMM = 26, concurrent acute PAMM = 2, concurrent AMN = 2) than in the control group (n = 4; all were resolved PAMM) (70.27% [95% CI: 53–84%] vs. 12.50% [95% CI: 4.1–30%], p < 0.001) (Table 1). BRVO and CRVO cases had similar prevalence of INL lesions in their fellow eyes (15/22, 68.1% vs. 11/15, 73.3%). Demographic and clinical data of individuals with and without resolved PAMM lesions are presented and compared in Table 2. Of people with resolved INL lesions, 86.67% (26/30) had RVO in their contralateral eye – 15 BRVO and 11 CRVO (Fig. 1). No significant difference regarding age, gender, background conditions, and history of IHD was noted (Table 2). The association between INL lesions presence and RVO (in the fellow eye) was statistically significant with an odds ratio of 15.7 (95%CI: 4.17–76.73, p < 0.001), unlike the association of RVO with DM (OR: 3.30, 95%CI: 0.85–15.89, p = 0.08), IHD (OR: 1.50, 95%CI: 0.44–5.35, p = 0.58), and gender (OR [male]: 2.66, 95%CI: 0.88–8.34, p = 0.08).

**Table 1:** Demographic and history information, visual acuity, and inner nuclear lesions presence in cases and controls

Characteristic	Overall (N = 69)		RVO group (N = 37)		Control group (N = 32)		p-value ^1^	q-value ^2^
		95% CI		95% CI		95% CI ^1^		
**Age**, mean (SD)	60.16 (7.75)		60.95 (8.43)	58–64	59.25 (6.90)	57–62	0.4	0.5
**Gender**, n (%)							0.08	0.15
***F***	26 (37.68%)	27–50%	10 (27.03%)	14–44%	16 (50%)	34–66%		
***M***	43 (62.32%)	50–73%	27 (72.97%)	56–86%	16 (50%)	34–66%		
**IHD**, n (%)	18 (26.09%)	17–38%	11 (29.73%)	16–47%	7 (21.88%)	9.9–40%	0.6	0.7
**DM**, n (%)	16 (23.19%)	14–35%	12 (32.43%)	19–50%	4 (12.50%)	4.1–30%	0.085	0.15
**VA LogMAR**, median (range)	0.046 (0.0–0.155)		0.097 (0.0–0.155)		0.046 (0.0–0.097)		< 0.001	< 0.001
**INL lesions presence**, n (%)	30 (43.48%)	32–56%	26 (70.27%)	53–84%	4 (12.50%)	4.1–30%	< 0.001	< 0.001

1- Welch Two Sample t-test; Mann-Whitney U test; Fisher’s exact test

2- False discovery rate correction for multiple testing

**Abbreviations**: DM, diabetes mellitus; F, female; IHD, ischemic heart disease; INL, inner nuclear layer; LogMAR, logarithm of minimum angle of resolution M, male; N, number; RVO, retinal vein occlusion; VA, visual acuity

**Table 2:** Comparison of eyes with and without evident inner nuclear layer lesions

Characteristic	With INL lesions (N = 30)		Without INL Lesions (N = 39)		p-value ^1^	q-value ^2^
		95% CI		95% CI ^1^		
**Group**, n (%)					< 0.001	< 0.001*
**RVO**	26 (86.67%)	68–96%	11 (28.21%)	16–45%		
**Control**	4 (13.33%)	4.4–32%	28 (71.79%)	55–84%		
**Age**, mean (SD)	62.27 (9.22)	59, 66	58.54 (6.03)	57, 60	0.061	0.1
**Gender**, n (%)					0.13	0.2
***F***	8 (26.67%)	13–46%	18 (46.15%)	30–63%		
***M***	22 (73.33%)	54–87%	21 (53.85%)	37–70%		
**IHD**, n (%)	10 (33.33%)	18–53%	8 (20.51%)	9.9–37%	0.3	0.3
**DM**, n (%)	9 (30%)	15–50%	7 (17.95%)	8.1–34%	0.3	0.3
**VA LogMAR**, median (range)	0.097 (0.0–0.155)		0.046 (0.0–0.155)		< 0.001	< 0.001
**RVO type**, n (%)					< 0.001	< 0.001*
*None*	4 (13.33%)	4.4–32%	28 (71.79%)	55–84%		
*BRVO*	15 (50%)	33–67%	7 (17.95%)	8.1–34%		
*CRVO*	11 (36.67%)	21–56%	4 (10.26%)	3.3–25%		

1- Welch Two Sample t-test; Mann-Whitney U test; Fisher’s exact test

2- False discovery rate correction for multiple testing

**Abbreviations**: BRVO, branch retinal vein occlusion; CRVO, central retinal vein occlusion; DM, diabetes mellitus; F, female; IHD, ischemic heart disease; INL, inner nuclear layer; LogMAR, logarithm of minimum angle of resolution M, male; N, number; RVO, retinal vein occlusion; VA, visual acuity

## Discussion

Our main findings were: (i) in a cohort of age-matched PwHTN, fellow eyes of RVO cases had INL lesions more frequently than control eyes, (ii) resolved INL lesions had a more pronounced association with RVO than other risk indicators, and (iii) CRVO and BRVO cases had similar frequencies of INL lesions in their fellow eyes.

Despite the wide range of identified predisposing factors, the development of RVO remains unpredictable in people with such factors. For example, although HTN has shown the most robust association with RVO, it occurs in only a small fraction of PwHTN [7–9]. Identifying early markers of excessive risk of RVO in people with established risk factors could enable earlier interventions to mitigate the risk of RVO.

Retinal ischemic lesions usually surface at perivenular sites and may remain confined or progress spatially [3, 11]. They may go unnoticed if small and localized further from the macula [11]. On cross-sectional OCT scans, atrophic INL lesions can be appreciated as foci of INL thinning and outer plexus layer (OPL) elevation – and as “black holes” on en face OPL slabs [5]. The association of resolved PAMM lesions with HTN, an established contributing factor to RVO, has been documented; Burnasheva et al. found that PwHTN (without RVO) were 40 times more likely than healthy people to have resolved PAMM [5]. A previous work by Maltsev and associates found resolved PAMM lesions in fellow eyes of people with RVO more frequently than in controls (71.2%, 47/66 vs. 19.3%, 11/45). Of note, both their cases and control groups were mixed and consisted of both hypertensive and normotensive individuals; resolved PAMM was not found in normotensive ones [11]. We included only PwHTN in each group, enabling us to form a relatively homogeneous cohort regarding their HTN status.

Our results suggested that INL ischemic lesions in PwHTN were associated with a 15-fold increase in their odds of developing RVO. Like Maltsev et al. [11], we found no difference between CRVO and BRVO regarding INL lesion prevalence in contralateral eyes. However, while only 12.5% (4/32) of PwHTN in our study had resolved PAMM lesions, works by Maltsev and colleagues [5, 11] reported higher frequencies (25.8%, 8/31 and 88.9%, 24/27, respectively). Intriguingly, the mean age of PwHTN in our study was about ten years older than those recruited by Burnasheva and colleagues [5]. Such discrepancy may be due to inter-study differences in average HTN severity. Unfortunately, our study was limited in that we did not measure the severity of HTN of our participants.

Some other limitations also apply to the present work, including the possibility of recall bias and lack of longitudinal data recording, which are common to case-control studies. Our sample size was sufficient to establish a link between RVO and INL lesions, but it lacked the power to reliably evaluate other risk factors with smaller effect sizes. Another limitation was that only one specialist examined the OCT scans for evidence of INL lesions. We also did not evaluate some other possible risk factors of RVO, such as ocular biometric parameters, duration of HTN, history of renal/cerebrovascular complications, thrombophilia, and so on. However, our main objective was to study the relationship between RVO and INL infarction on OCT, as a subclinical sequela of various risk factors and pathomechanisms, which may ultimately result in RVO. Thus, an INL ischemic lesion was assumed as an *indicator* of increased risk, rather than a causative factor among others.

To conclude, utilizing structural OCT, we identified a substantially higher prevalence of INL lesions in PwTHN with RVO compared to PwHTN without RVO. The atrophic appearance of lesions suggests they may serve as early markers of increased RVO risk in individuals with systemic or cardiovascular predisposing factors. Upon detecting active or atrophic INL lesions in an otherwise seemingly healthy eye, it is important to (i) be concerned about potentially severe forthcoming events, (ii) inform patients about their high-risk profile, (iii) advise them to adopt more stringent lifestyle modifications, and (iv) consider consulting with internal medicine and cardiology specialists.

## Data Availability

The data supporting the findings of this study are available from the corresponding authors (HN and SHA) upon reasonable request.

## References

[1] Khayat M, Williams M, Lois N. Ischemic retinal vein occlusion: characterizing the more severe spectrum of retinal vein occlusion. Surv Ophthalmol. 2018 Nov;63(6):816–50. 10.1016/j.survophthal.2018.04.005 29705175

[2] Schmidt-Erfurth U, Garcia-Arumi J, Gerendas BS, Midena E, Sivaprasad S, Tadayoni R (2019). Guidelines for the management of retinal vein occlusion by the european society of retina specialists (EURETINA). Ophthalmol J Int Ophtalmol Int J Ophthalmol Z Augenheilkd.

[3] Abtahi SH, Nourinia R, Mazloumi M, Nouri H, Arevalo JF, Ahmadieh H. Retinal ischemic cascade: new insights into the pathophysiology and imaging findings. Surv Ophthalmol. 2022 Dec 2;S0039-6257(22)00173-4. 10.1016/j.survophthal.2022.11.009 36464134

[4] Bakhoum MF, Freund KB, Dolz-Marco R, Leong BCS, Baumal CR, Duker JS, et al. Paracentral Acute Middle Maculopathy and the ischemic Cascade Associated with Retinal vascular occlusion. Am J Ophthalmol. 2018 Nov;195:143–53. 10.1016/j.ajo.2018.07.031 30081014

[5] Burnasheva MA, Maltsev DS, Kulikov AN, Sherbakova KA, Barsukov AV. Association of Chronic Paracentral Acute Middle Maculopathy Lesions with Hypertension. Ophthalmol Retina. 2020 May;4(5):504–9. 10.1016/j.oret.2019.12.001 31948908

[6] Sjoberg DD, Whiting K, Curry M, Lavery JA, Larmarange J (2021). Reproducible Summary tables with the gtsummary Package. R J.

[7] Song P, Xu Y, Zha M, Zhang Y, Rudan I. Global epidemiology of retinal vein occlusion: a systematic review and meta-analysis of prevalence, incidence, and risk factors. J Glob Health. 2019 Jun;9(1):010427. 10.7189/jogh.09.010427 PMC6513508 31131101

[8] Wang Q, Liu L, Jonas JB, Gao B, Wu SL, Chen SH et al. Albuminuria and retinal vessel density in diabetes without diabetic retinopathy: the Kailuan Eye Study. Acta Ophthalmol (Copenh). 2021 Aug;99(5):e669–78. 10.1111/aos.14670 33354932

[9] Yasuda M, Kiyohara Y, Arakawa S, Hata Y, Yonemoto K, Doi Y, et al. Prevalence and systemic risk factors for retinal vein occlusion in a general japanese population: the Hisayama study. Invest Ophthalmol Vis Sci. 2010 Jun;51(6):3205–9. 10.1167/iovs.09-4453 20071683

[10] Scharf J, Freund KB, Sadda S, Sarraf D. Paracentral acute middle maculopathy and the organization of the retinal capillary plexuses. Prog Retin Eye Res. 2021 Mar;81:100884. 10.1016/j.preteyeres.2020.100884 32783959

[11] Maltsev DS, Kulikov AN, Burnasheva MA, Chhablani J. Prevalence of resolved paracentral acute middle maculopathy lesions in fellow eyes of patients with unilateral retinal vein occlusion. Acta Ophthalmol (Copenh) [Internet]. 2020 Feb [cited 2023 Jan 7];98(1). Available from: https://onlinelibrary.wiley.com/doi/10.1111/aos.14196. 10.1111/aos.14196 31347293

